# Prediction of Outcome from Community-Acquired Severe Sepsis and Septic Shock in Tertiary-Care University Hospital in a Developing Country

**DOI:** 10.1155/2012/182324

**Published:** 2012-10-17

**Authors:** Krsto Grozdanovski, Zvonko Milenkovic, Ilir Demiri, Katerina Spasovska

**Affiliations:** Department of Intensive Care, University Clinic for Infectious Diseases, Vodnjanska 17, 1000 Skopje, Macedonia

## Abstract

Our aim was to determine the risk factors on mortality in adult patients with community-acquired severe sepsis and septic shock. The main outcome measure was hospital mortality. This prospective single centre study was conducted from January 1, 2008 to December 31, 2010, and included 184 patients, of whom 135 (73.4%) were with severe sepsis and 49 (26.6%) had septic shock. Overall, ninety-five (51.6%) patients have died, 60 (44.4%) in severe sepsis and 35 (71.4%) patients with septic shock. The lung was the most common site of infection 121 (65.8%), and chronic heart failure was the most frequent comorbidity 65 (35.3%). Logistic multivariate analysis identified three independent risk factors for mortality in patients with severe sepsis: positive blood culture (odds ratio, 2.39; 95% confidence interval, 1.13–5.06; *P* = 0.02), three or more organ dysfunctions (odds ratio, 3.93; 95% confidence interval, 1.62–9.53; *P* = 0.002), and Simplified Acute Physiology Score (SAPS) II (odds ratio, 1.02; 95% confidence interval, 1.00–1.04; *P* = 0.01). In addition to SAPS II, positive blood culture, and three or more organ dysfunctions are important independent risk factors for mortality in patients with severe sepsis and septic shock.

## 1. Introduction

Severe sepsis is a clinical syndrome defined as an infection complicated by acute organ dysfunction [1, 2]. Even in developed countries, severe sepsis is very common, the treatment is costly and is one of the leading causes of death [3]. A recent study conducted in the United States showed increase in the number of hospitalized cases of severe sepsis from 415,280 in 2003 to 711,736 in 2007 which is a 71% increase, with an annual growth rate of 17.8% per year. The same study indicates that from 2003 to 2007 the proportion of patients with three, four, or more organ dysfunctions significantly have increased. Regardless of more complicated course of severe sepsis, hospital mortality is declining from 37% in 2003 to 29% in 2007 [4]. Data from the SOAP study, performed mostly in developed European countries reported an ICU mortality of 32.2% in patients with severe sepsis, 54.1% in cases with septic shock, and 65% in patients with four or more organ failures [5].

However, most information about severe sepsis and septic shock are based on mixed population including both community and hospital-acquired sepsis. There are a limited number of studies focused only on patients with community-acquired sepsis [6–8].

In addition, data regarding different aspects of severe sepsis and septic shock in developing countries especially from the Balkan region are scarce. Thus, the main objective of this study was to determine specific risk factors for mortality in patients with community-acquired severe sepsis and septic shock. 

## 2. Materials and Methods

### 2.1. Study Overview

This was a prospective observational study conducted in a 6-bed medical ICU at the University Clinic for Infectious Diseases, a 130-bed tertiary-care hospital in Skopje, Republic of Macedonia (Former Yugoslav Republic of Macedonia). The study was approved by the local hospital institutional ethics committee and took place from January 1, 2008 to December 31, 2010. All new admissions aged ≥ 18 years with community-acquired severe sepsis or septic shock were assessed for possible enrollment in the study. Patients were not included if sepsis was a result of nosocomial infection or if transferred from another hospital or if patients were residing in rehabilitation centers or nursing homes, since they were classified as having healthcare-associated sepsis. The exclusion criteria were a stay in the ICU less than 24 hrs, noninfectious cause of systemic inflammation, or if immediate surgery was required.

For each patient, at admission, we recorded demographic data and comorbid medical conditions. Clinical and laboratory data for Simplified Acute Physiology Score (SAPS) II [9] were collected within 24 hours of admission. Data for source of infection, number of organ dysfunctions, and values of inflammatory markers (C-reactive protein, white blood cells, and erythrocyte sedimentation rate) were evaluated on a daily basis. All patients had two blood cultures drawn from separate venipuncture sites at admission to the ICU. Blood cultures obtained after this period were not considered for analysis.

Infection sites were categorized as one of the following: lower respiratory tract, meningeal, soft tissue, urinary tract, abdominal, endocarditis, and unknown.

Patients were followed until discharge or until death in the hospital. The primary outcome measure was hospital mortality.

### 2.2. Definitions

Infection was defined as a pathologic process caused by the invasion of normal sterile tissue, fluid or body cavity by a pathogenic or potentially pathogenic microorganism (not believed to be a contaminant) and/or clinically suspected infection [1, 2]. Community-acquired infection was defined as infection manifesting before or within 48 hours after ICU admission.

Sepsis was defined as infection plus at least two systemic inflammatory response syndrome criteria. Severe sepsis was defined by sepsis plus at least one sepsis-induced acute organ dysfunction. Organ dysfunction was defined as follows: cardiovascular system failure was systolic blood pressure <90 mm Hg or mean arterial blood pressure <70 mm Hg or drop in systolic blood pressure >40 mm Hg from baseline; renal dysfunction was urinary output ≤700 mL/day or serum creatinine >176.8 *μ*moL/L; respiratory dysfunction was PaO_2_ <70 mm Hg or mechanical ventilation or PaO_2_/FiO_2_ ≤250 (or ≤200 in patients with pneumonia); thrombocytopenia was defined as platelet count <80,000/mm^3^; hepatic dysfunction was hyperbilirubinemia (plasma total bilirubin >34.2 *μ*moL/L) or a threefold increase in serum aminotransferases; acidosis pH ≤7,3 or base excess ≥5 mmoL/L; central nervous system (CNS) dysfunction was Glasgow coma scale <13. Septic shock was defined when sepsis resulted in arterial hypotension needing vasopressors, despite initial adequate fluid resuscitation [1, 2].

### 2.3. Statistical Analysis

Kolmogoroff-Smirnov test was used to verify the normality of distribution of continuous variables. Normally distributed variables are presented as mean (SD) and nonnormally distributed variables as median (1st quartile-3rd quartile). Difference testing between groups was performed using the Student's *t*-test when data were normally distributed. When normality was rejected nonparametric Mann-Whitney *U*-test was used for independent groups. Categorical variables were expressed as numbers and percentages and analyzed using the chi-square and Fisher exact test when necessary. All potentially relevant variables (*P* < 0.05) on univariate analysis were entered into the multivariate analysis. A multivariate stepwise logistic regression analysis with the hospital mortality as the dependent factor was performed in order to identify independent predictors of outcome in patients with severe sepsis. All statistics were two-tailed, and a *P* < 0.05 was considered to be significant. Data were analyzed with SPSS 17.0 software (SPSS, Chicago, IL).

## 3. Results

During the study period, a total of 875 patients were admitted into the ICU of whom 268 had a diagnosis of severe sepsis or septic shock. Twenty-one patients were excluded from the study because they were younger than 18 years of age, 19 had noninfectious cause of SIRS and organ dysfunction, 39 patients were excluded for staying in ICU less than 24 hours, 5 were transferred to surgery. A total of 184 (21%) patients met inclusion criteria for community-acquired severe sepsis and were included in the analysis. All patients had one episode of sepsis. Severe sepsis and septic shock were identified in 135 (73.4%) and 49 (26.6%) patients, respectively. The overall mortality rate was 51.6% (*n* = 95). The hospital mortality of patients with severe sepsis was 44.4% (*n* = 60) and 71.4% (*n* = 35) in those with septic shock.

A summary of demographics, comorbidities, severity, sources, and number of organ dysfunction are shown in Table 1. The patients mean age was 57.1 years (SD 17.9); 122 (66,3%) were male. The majority of patients 136 (73.9%) had one or more comorbid conditions. Higher mortality was observed in patients with three or more comorbidities and in those with chronic heart failure (*P* = 0.004) or chronic respiratory failure (*P* = 0.001).

Lower respiratory tract infections were identified as the most frequent source of infection in 121 (65.8%), followed by meningitis in 17 (9.2%) and skin/soft tissues infections in 15 (8.2%) cases (Table 1).

The most common organs with acute dysfunction were acute respiratory failure 101 (54.9%), hematologic disorder 89 (48.4%), and renal dysfunction 81 (44.0%).

Results of analyzed inflammatory markers did not show significant difference between survivors and nonsurvivors (Table 1).

Blood cultures were positive in 63 (34.2%) patients. Among them, Gram-positive microorganisms were isolated in 49 (77.8%) and Gram-negatives in 14 (22.2%). *Staphylococcus aureus* accounted for 59.2% (*n* = 29) of Gram-positive isolates, followed by *Streptococcus pneumoniae* 22.4% (*n* = 11). Analysis showed that the finding of a positive blood culture is related to mortality (*P* = 0.008), regardless of the Gram stain results or the type of isolated organism (Table 2).

Univariate analysis identified several factors associated with significantly higher mortality in patients with sepsis: age (*P* = 0.01), number of comorbidities (*P* = 0.02), chronic heart failure (*P* = 0.004), chronic respiratory failure (*P* = 0.001), number of failed organs (*P* < 0.001), septic shock (*P* = 0.001), positive blood culture (*P* = 0.008), and SAPS II score (*P* < 0.001).

Three or more failing organs, positive blood culture, and SAPS II score were determined as independent predictors of mortality in patients with severe sepsis by multivariate analysis using logistic regression (Table 3).

## 4. Discussion

Our study found a high number of admissions with community-acquired severe sepsis and septic shock (21%) to the ICU. Data on severe sepsis incidence vary between studies and countries performed. In developed European region it ranges from 10.5% in Finland [10], 14.6% in French ICUs [11] to 66.5% in a British ICU [12]. The data from developing countries are also variable ranging from 8.7% in China [13], 18.9% in Thailand [14] to 51% in Colombia [15]. A recent Portuguese study showed occurrence of community-acquired sepsis of 22%, which is in accordance with our observation [16].

In our series 66.3% of the patients were male but gender did not influence the outcome, a finding supported by other studies as well [3, 11, 17]. Age has been associated with increased mortality rate in severe sepsis patients [5, 18]. In our study nonsurvivors were older than survivors, however age was not identified as an independent predictor of mortality which was in agreement with other reports [11, 19].

The overall mortality rate in our study was 51.6%. The mortality in patients with severe sepsis was 44.4%, and a very high mortality of 71.4% in patients with septic shock. Several studies report similar mortality rate in patients with community-acquired severe sepsis as our findings [6, 7]. A study from a tertiary care hospital in Turkey found mortality in patients with community-acquired severe sepsis of 67.7% and 91.2% mortality in patients with hospital-acquired sepsis [20]. A Spanish study reported a hospital death of 52.1% in patients with community-acquired sepsis [21]. Contrary to these reports, in his recent study, Zahar and the team showed 24.6% and 40.5% mortality from community-acquired severe sepsis and septic shock, respectively [22]. The Portuguese study presented even lower results of mortality from severe sepsis of 19% and 44% from septic shock [16]. The SOAP study reported a mortality rate of 32.2% for patients with severe sepsis and 54.1% for patients with septic shock keeping in mind that this study was not focused only on community-acquired sepsis [5]. Our results compare unfavorably with the recent trends especially from highly industrialized countries where mortality from sepsis is decreasing every year by 2% despite the severity of illness that is increasing [4]. A possible explanation for high mortality in our study is due to the poor compliance to the Surviving Sepsis Campaign recommendations [23], mainly in the part of fluid resuscitation, especially now when we have clear evidence that improved compliance to sepsis bundles is associated with decreased mortality [23–25]. As a result of limited resources in our setting we are not always able to provide central venous catheters, central venous pressure monitoring, and central venous oxygen saturation, to measure lactates and to administer vasopressors. Septic shock was clearly associated with mortality in univariate analysis (*P* = 0.001) but did not appear as an independent risk factor for mortality in multivariate analysis, possibly because of influence of SAPS, and low number of patients with septic shock included in the analysis. Other studies report much higher number of patients with community-acquired septic shock from 34% to 74.1% [6, 22].

The lung proved to be the most common site of infection in our study (65.8%), a finding consistent with many other reports [5, 12, 19]. However, as in other published studies regarding community-acquired sepsis, we did not find an influence of the source of infection on patients' mortality [6, 7, 22].

Chronic heart failure appear to be associated with the risk of death [11, 19]. Our study did not find chronic heart failure as independent risk factor for hospital mortality, which is in accordance with a Spanish study of community-acquired sepsis [6].

Two blood cultures from two separate sites were obtained from all 184 patients from whom 34.2% were positive. In our study Gram-positive bacteria were predominantly isolated which differs from the reports showing a trend towards predominance of Gram-negative microorganisms [22, 26]. However studies focused on community-acquired sepsis showed similar data, as our observation of a predominance of Gram-positives [6, 7]. We found that a positive blood culture contributes to patients' outcomes which is in concordance with some studies [27, 28], but the type of organism and site of infection were not associated with increased risk of death which is in agreement with recent reports [6, 7, 12, 22].

Our results are consistent with previous reports of prognostic factors in severe sepsis and septic shock, where the severity index score SAPS II was found to be associated with a poor outcome [11, 19, 27].

This observational study has several limitations. As a single-centre study it may not reflect the trends of other medical ICUs in the country. Also we did not evaluate ICU and hospital-acquired sepsis, patients pretreatment, as well as appropriateness of antimicrobial therapy and early goal-directed therapy. Relatively low number of patients and limited interventions in our settings might influence the results. Thus a multicentre study is envisaged to provide more accurate data for the whole country. Further research on severe sepsis and septic shock that will include the treatment interventions to patient outcome should be considered.

In conclusion, severe sepsis is a frequent cause of ICU admission with high mortality especially for patients with septic shock. Early identification of factors predicting mortality is crucial for aggressive and appropriate management of the patients. Our results highlight the importance of SAPS II score, positive blood culture, and three or more organ dysfunctions as independent factors associated with mortality. This will benefit the early identification of patients at high risk for poor outcomes that contributes to intensive management and appropriate treatment interventions.

## Figures and Tables

**Table 1:** Demographic and clinical characteristics of patients with severe sepsis according to outcome.

Variable	All patients (*n* = 184)	Survivors (*n* = 89, 48.4%)	Nonsurvivors (*n* = 95, 51.6%)	*P* value
Male, *n* (%)	122 (66.3)	56 (63.0)	66 (69.5)	0.34
Female, *n* (%)	62 (33.7)	33 (37.0)	29 (30.5)	
Age, mean (SD)	57.1 (17.9)	53.7 (18.3)	60.3 (17.1)	0.01
Hospital stay, days, median (Q1–Q3)	13 (6–20)	18 (13.5–28)	6 (4–12)	<0.001
Number of comorbidities, *n* (%)				
0	48 (26.1)	30 (33.7)	18 (18.9)	0.02
1	76 (41.3)	33 (37.1)	43 (45.3)	
2	42 (22.8)	22 (24.7)	20 (21.1)	
≥3	18 (9.8)	4 (4.5)	14 (14.7)	
Comorbidities				
Chronic heart failure	65 (35.3)	22 (24.7)	43 (45.3)	0.004
Diabetes mellitus	38 (20.7)	18 (20.2)	20 (21.1)	0.89
Hemiplegic stroke	28 (15.2)	13 (14.6)	15 (15.8)	0.82
Chronic respiratory failure	22 (12.0)	3 (3.4)	19 (20.0)	0.001
Chronic alcoholism	19 (10.3)	11 (12.4)	8 (8.4)	0.38
Metastatic cancer	14 (7.6)	8 (9.0)	6 (6.3)	0.49
Chronic renal failure	12 (6.5)	4 (4.5)	8 (8.4)	0.28
Chronic liver failure	12 (6.5)	7 (7.9)	5 (5.3)	0.47
SAPS II score, median (Q1–Q3)	35 (21–53)	30 (18–37)	43 (25–64)	<0.001
Site of infection, *n* (%)				
Lower respiratory tract	121 (65.8)	56 (62.9)	65 (68.4)	0.17
Meningitis Soft tissue	17 (9.2) 15 (8.1)	8 (9.0) 6 (6.7)	9 (9.5) 9 (9.5)	
Urinary tract	10 (5.4)	9 (10.1)	1 (1.0)	
Abdominal (nonsurgical)	6 (3.3)	2 (2.3)	4 (4.2)	
Endocarditis	9 (4.9)	4 (4.5)	5 (5.3)	
Unknown	6 (3.3)	4 (4.5)	2 (2.1)	
Number of failed organs, *n* (%)				
1	61 (33.2)	45 (50.6)	16 (16.8)	<0.001
2	53 (28.8)	25 (28.1)	28 (29.5)	
≥3	70 (38.0)	19 (21.3)	51 (53.7)	
Septic shock, *n* (%)	49 (26.6)	14 (15.7)	35 (36.8)	0.001
Laboratory results				
ESR, mm/h, mean (SD)	67.9 (32.6)	70.8 (33.5)	63.9 (31.5)	0.40
WBC × 10^9^/L, mean (SD)	15.9 (9.1)	15.1 (9.0)	16.8 (9.2)	0.19
CRP mg/L, mean (SD)	313.5 (183.9)	330.1 (186.6)	297 (180.8)	0.21

SD: standard deviation; Q1–Q3: 1st quartile–3rd quartile; SAPS II: simplified acute physiology score II; ESR: erythrocyte sedimentation rate; WBC: white blood cells; CRP: C-reactive protein.

**Table 2:** Microorganisms isolated from blood cultures in patients with severe sepsis and septic shock according to outcome.

Microorganism	All patients (*n* = 184)	Survivors (*n* = 89, 48.4%)	Nonsurvivors (*n* = 95, 51.6%)	*P* value
Blood culture positive, *n* (%)	63 (34.2)	22 (24.7)	41 (43.2)	0.008
Gram-positive, *n* (%)	49 (26.6)	18 (20.2)	31 (32.6)	0.36
Gram-negative, *n* (%)	14 (7.6)	4 (4.5)	10 (10.5)	
Gram-positive, *n* (%)				
*Staphylococcus aureus *	29 (15.8)	12 (13.5)	17 (17.9)	0.33
*Streptococcus pneumoniae *	11 (6.0)	4 (4.5)	7 (7.4)	
*Enterococcus *species	6 (3.3)	1 (1.1)	5 (5.3)	
*Listeria monocytogenes *	2 (1.1)	1 (1.1)	1 (1.1)	
*Streptococcus viridans *	1 (0.5)	0 (0)	1 (1.1)	
Gram-negative, *n* (%)				
*Escherichia coli *	6 (3.3)	2 (2.2)	4 (4.2)	
*Klebsiella pneumoniae *	3 (1.6)	1 (1.1)	2 (2.1)	
*Pseudomonas aeruginosa *	3 (1.6)	1 (1.1)	2 (2.1)	
*Proteus mirabilis *	2 (1.1)	0 (0)	2 (2.1)	

**Table 3:** Multivariate logistic regression analysis with hospital mortality as the dependent factor.

Parameter	OR (95% CI)	*P* value
Positive blood culture	2.39 (1.13–5.06)	0.02
≥3 failed organs	3.93 (1.62–9.53)	0.002
SAPS II score	1.02 (1.00–1.04)	0.01

OR: odds ratio; CI: confidence interval; SAPS II: simplified acute physiology score II.
